# High prevalence of vitamin D insufficiency and its association with BMI-for-age among primary school children in Kuala Lumpur, Malaysia

**DOI:** 10.1186/1471-2458-11-95

**Published:** 2011-02-11

**Authors:** Geok L Khor, Winnie SS Chee, Zalilah M Shariff, Bee K Poh, Mohan Arumugam, Jamalludin A Rahman, Hannah E Theobald

**Affiliations:** 1 Department of Nutrition and Dietetics, School of Pharmacy & Health Sciences, International Medical University, Bukit Jalil, 57000 Kuala Lumpur, Malaysia; 2 Department of Nutrition and Dietetics, Faculty of Medicine & Health Sciences, Universiti Putra Malaysia, 43400 Serdang, Malaysia; 3 Department of Nutrition and Dietetics, Faculty of Allied Health Sciences, Universiti Kebangsaan Malaysia, 50300 Kuala Lumpur, Malaysia; 4 Department of Medicine, Serdang Hospital, 43000 Kajang, Malaysia; 5 Department of Community Health and Family Medicine, Faculty of Medicine, International Islamic University Malaysia, 25200 Kuantan, Malaysia; 6 GlaxoSmithKline Nutritional Healthcare, 980 Great West Road, Brentford, TW8 9GS, UK

## Abstract

**Background:**

Deficiencies of micronutrients can affect the growth and development of children. There is increasing evidence of vitamin D deficiency world-wide resulting in nutritional rickets in children and osteoporosis in adulthood. Data on the micronutrient status of children in Malaysia is limited. The aim of this study was to determine the anthropometric and micronutrient status of primary school children in the capital city of Kuala Lumpur.

**Methods:**

A cross sectional study of primary aged school children was undertaken in 2008. A total of 402 boys and girls aged 7-12 years, attending primary schools in Kuala Lumpur participated in the study. Fasting blood samples were taken to assess vitamin D [as 25(OH)D], vitamin B_12_, folate, zinc, iron, and ferritin and haemoglobin concentrations. Height-for-age and body mass index for age (BMI-for-age) of the children were computed.

**Results:**

Most of the children had normal height-for-age (96.5%) while slightly over half (58.0%) had normal BMI-for-age. A total of 17.9% were overweight and 16.4% obese. Prevalence of obesity was significantly higher among the boys (25%) than in the girls (9.5%) (χ^2 ^= 22.949; *P *< .001). Most children had adequate concentrations of haemoglobin, serum ferritin, zinc, folate and vitamin B_12_. In contrast, 35.3% of the children had serum 25(OH)D concentrations indicative of vitamin D deficiency(≤37.5 nmol/L) and a further 37.1% had insufficiency concentrations (> 37.5-≤50 nmol/L). Among the boys, a significant inverse association was found between serum vitamin D status and BMI-for-age (χ^2 ^= 5.958; *P *= .016).

**Conclusions:**

This study highlights the presence of a high prevalence of sub-optimal vitamin D status among urban primary school children in a tropical country. In light of the growing problem of obesity in Malaysian children, these findings emphasize the important need for appropriate interventions to address both problems of obesity and poor vitamin D status in children.

## Background

Micronutrient deficiencies are common world-wide and adversely affect growth, health, behavioural and cognitive development in children [1]. Serious micronutrient deficiencies may lead to death. It is estimated that deficiencies of vitamin A and zinc were responsible for 0.6 million and 0.4 million child deaths, respectively in 2008 [2]. Thus, the importance of micronutrient interventions to reduce morbidity and mortality in children is well recognized.

In Malaysia, data on the micronutrient status of children is derived mostly from dietary studies. Such studies face methodological challenges including poor availability of data on the content of key micronutrients in local foods. There is a scarcity of studies on micronutrients in young children determined through urine or blood analysis [3]. There are a few such studies at the national level undertaken mainly by the Ministry of Health (MOH). These include a study on iodine deficiency disorders (IDD) among children aged 8-10 years in 2008, which showed that the majority of the children had normal urinary iodine excretion concentrations (> 100 μg/L), but 31.6% had mild IDD levels and another 14.2% with moderate severity status [4]. As for vitamin A deficiency, Malaysia is considered to have a mild public health problem by the World Health Organization [5], based on the MOH/UNICEF study in 1999-2000, which reported a national prevalence of vitamin A deficiency of 3.5% (serum retinol <0.70 μmol/L) among preschool-aged children. Anaemia is a common problem in young children from poor communities and in the last decade or so, levels of 20-23% and 16-17% respectively in children aged 7-12.9 years and 13-18 years from rural areas were reported [6].

In contrast, there is copious anthropometric data on children across Malaysia. The largest national assessment of the nutritional status of children is the Third National Health and Morbidity Survey (NHMS III) conducted in 2006, involving more than 21,000 children below 18 years of age [7]. The NHMS III reported underweight in 13.2%, stunting in 15.8% and overweight in 5.4%. These data indicate markedly lower levels of under-nutrition in Malaysian children compared to previous studies and unpublished MOH national surveillance data. However, overweight prevalence is increasing in recent years. In 2001-2002, a study on about 12,000 schoolchildren aged 6-12 years in Peninsular Malaysia reported that 10.5% were overweight and 5.9% obese [8. For the same age group, the NHMS III in 2006 reported overweight and obesity as 15.9% and 12% respectively [7].

Against the backdrop of limited data on micronutrient status, and evidence of increasing overweight in children, this study was undertaken to determine the status of key micronutrients based on biochemical analysis in children and to relate the findings to their nutritional status.

## Methods

### Subjects and sampling

This cross-sectional study was carried out amongst boys and girls aged 7-12 years attending primary schools in Kuala Lumpur, the capital city of Malaysia. Primary schools in Kuala Lumpur are classified into four zones and the Pudu zone was purposively selected for its mixture of ethnicity and income levels. Among those schools with more than 1000 pupils, five schools were randomly selected to participate in the study.

An invitation to participate in the study was sent out to parents and guardians of all children within the targeted age-group from the selected primary schools. Exclusion criteria were: chronic or recent (previous two weeks) illness; the following of special or restrictive diets and being of non-Malaysian nationality. Parental consent and the child's assent were required from all subjects.

Using the reported prevalence of anaemia in urban children as the basis on which to calculate the required sample size, a total sample size of 400 primary school children (200 boys and 200 girls) was deemed as being sufficient to estimate the prevalence of anaemia to the order of 15%, to within 3% with 95% confidence.

### Blood analyses

The subjects were requested to fast for at least 10 hours before they came for the study. A team comprising phlebotomists, researchers and an attendant physician was present during each session of taking blood samples and anthropometric measurements. The fasting blood samples, totalling 10 mL were drawn into Vacutainers (8 mL into a plain tube and a 2 mL into an EDTA tube) (Beckton Dickinson & Company, Franklin Lakes, US) through venepuncture to assess serum micronutrients. Immediately upon collection, samples were wrapped in aluminium foil (to prevent access by UV light to the sample) and stored on ice. Samples were centrifuged to separate serum at the end of each daily collection and serum aliquoted off and stored at -20°C until analysed.

Micronutrients determined in this study were selected for their associations with anaemia and cognitive development in school-aged children. Ferritin, folate and vitamin B_12 _were determined by automated competitive immunoassay (Advia 2400 Chemistry Systems, Siemans, USA); iron by automated spectrometry (Advia 2400 Chemistry Systems, Siemans, USA), and full blood count (haemoglobin, haematocrit, red cell count, mean corpuscular volume white cell count) by automated analyzer (Advia 120 Hematology System, Siemans, USA). Zinc was determined by Flame Atomic Absorption Spectrometry (AAS), while vitamin D status, as determined through the measurement of 25-hydroxyvitamin D or 25(OH)D, was analysed using the Diaspora Liason 25OH Vitamin D platform (Diasorin Inc, USA). All the biochemical analyses were outsourced to a private laboratory with international quality control Certificate of Accreditation (ISO 15189).

References used for the classification of serum micronutrient status are indicated as follows: haemoglobin concentration and mean corpuscular volume [9]; ferritin concentration [10]; zinc concentration [11]; vitamin B_12 _and folate concentrations [12]; vitamin D concentration [13].

### Anthropometric measurements

Participant's height and weight were measured in minimal light indoor clothing and without shoes. Body weight was measured to the nearest 0.1 kg using a digital lithium weighing scale (Tanita 318, Japan). Height, without shoes, was measured to the nearest 0.1 cm using a SECA portable body meter (SECA 206, Germany). Anthropometric status of the children was classified based on the World Health Organisation recommendation [14], using height-for-age, weight-for-age and BMI-for-age measurements.

### Statistical analysis

Data were analysed using PASW Statistics 18 (SPSS Inc., 2009, Chicago, Illinois) and Stata 10 (StataCorp. 2007. Stata Statistical Software: Release 10. College Station, TX: StataCorp LP). Categorical data were described using count, percentages and 95% confidence intervals when applicable. Numerical data were checked for normal distribution and described as mean and standard deviation if normally distributed and as median and inter-quartile range if not normally distributed. The chi-square test was used to examine the association between the categorical data on serum micronutrient concentrations and sex of the children. The significance level was set at p < 0.05.

### Ethical approval

Approval to conduct the study was granted by the schools, the Ministry of Education and the Department of Education of the Federal Territory of Kuala Lumpur. Ethical approval was obtained from the Medical Research Ethics Committee of the Faculty of Medicine and Health Sciences, Universiti Putra Malaysia.

## Results

### Demographic and socio-economic background

Out of approximately 2000 invitation letters accompanied by information sheets and consent forms that were distributed to the selected schools, the final number of children who participated with parental/guardian consent was 402, thereby giving a response rate of about 20%. The low response rate is attributed mainly to concern by parents/guardians and fear of children for drawing of blood (10 mL).

Among the 402 children who participated, there were almost equal proportions of those aged 7-9 years (48.3%) and 10-12 years (51.7%) (Table 1). The percentage of boys (44.8%) was somewhat lower than that of girls (55.2% girls). Their mean (SD) age was 9.9 (1.2) years. About half of the children were of Malay ethnicity (51.2%) followed by 42.8% Chinese and 6% Indian. The ethnic composition of the study children may be deemed typical of Malaysian school children, given that four national schools and a Chinese-medium school were among the five schools selected for the study.

**Table 1:** Distribution of children according to socio-demographic background (n = 402)

	N (%)	Mean ± SD
Boys	180 (44.8)	

Girls	222 (55.2)	

**Age (boys & girls)**		

7-12 years	402 (100)	9.9 ± 1.2

7-9 years	194 (48.3)	9.0 ± 0.9

10-12 years	208 (51.7)	10.9 ± 0.6

**Ethnicity**		

Malay	206 (51.2)	

Chinese	172 (42.8)	

Indian & others	24 (6.0)	

**Number of siblings (n = 401)**		3.6 ± 1.6

≤3	221 (55.1)	

≥4	180 (44.9)	

**Father's age (years) (n = 388)**		43.0 ± 6.2

≤ 40	139 (35.9)	

> 40	249 (64.1)	

**Mother's age (years) (n = 393)**		39.6 ± 5.6

≤ 40	226 (57.5)	

> 40	167 (42.5)	

**Father's educational attainment (n = 387)**		

Secondary school and below	270 (69.8)	

College/University	117 (30.2)	

**Mother's educational attainment (n = 389)**		

		

Secondary school and below	290 (74.6)	

College/University	99 (25.4)	

**Monthly household income (n = 391) (US$1.00 = RM3.00)**		

< RM3999	245 (62.7)	

RM4000 - RM7999	92 (23.5)	

≥ RM8000	54 (13.8)	

Family socio-demographics showed that the mean number of siblings in the families was 3.6 (1.6) and that the child studied was, on average, second or third in the birth order. The mean age of the fathers was 43.0 (6.2) years and that of the mothers was 39.6 (5.6) years. Approximately one-third of the fathers and one-quarter of the mothers had attained college/university education. While nearly half of the mothers were housewives, majority of the fathers were employed in professional, technical and managerial positions. A higher proportion of parents in this study had tertiary education than the educational attainment in the general labour force [15]. Overall, almost two-thirds of the households earned less than RM4000 a month, while a quarter had monthly earnings between RM4000-RM7999, thus placing the study households in the middle-income group (the current exchange rate is US$1.00 = RM3.00).

### Nutritional status

Most of the children (96.5%) had normal height-for-age (Table 2). However, only slightly over half (58.0%) had normal BMI-for-age, with a higher prevalence of the girls (65.3%) than boys (48.9%) having normal BMI-for-age. A total of 17.9% of the children were overweight while another 16.4% were obese. While nearly as many boys and girls were overweight, a significantly higher proportion of the boys was obese (25.0%) compared to the girls (9.5%) (χ^2 ^= 22.949; *P *< .001). A relatively small percentage of the children was too thin for their age (8.9% and 6.8% of the boys and girls, respectively).

**Table 2:** Distribution of children according to height-for-age and BMI-for-age

	Boys (N = 180)				Girls (N = 222)				Total (N = 402)			
	
			95% CI				95% CI				95% CI	
									
	%	SE	Lower	Upper	%	SE	Lower	Upper	%	SE	Lower	Upper
**Height-for-age**												

Normal	95.6	1.54	92.52	98.60	97.3	1.09	95.15	99.45	96.5	0.92	94.72	98.32

Stunted	4.4	1.54	1.40	7.48	2.7	1.09	0.55	4.85	3.5	0.92	1.68	5.28

**BMI-for-age**												

Severe thinness	2.8	1.23	0.35	5.20	0.5	0.45	0.44	1.34	1.5	0.61	0.30	2.68

Thinness	6.1	1.79	2.58	9.64	6.3	1.64	3.08	9.53	6.2	1.21	3.85	8.59

Normal	48.9	3.74	41.52	56.26	65.3	3.20	59.01	71.63	58.0	2.47	53.11	62.81

Overweight	17.2	2.82	11.65	22.79	18.5	2.61	13.32	23.61	17.9	1.91	14.15	21.67

Obese	25.0	3.24	18.61	31.39	9.5	1.97	5.58	13.34	16.4	1.85	12.78	20.05

Height-for-age vs sex of the children: χ2 = 0.897, df = 1, p = 0.344 (Monte Carlo)

BMI-for-age vs sex of the children: χ2 = 22.949, df = 4, p < 0.001 (Monte Carlo)

Stunting was found in only 3.5% of the children, being somewhat higher among the boys (4.4%) than in the girls (2.7%). This result indicates that most of the children who were overweight or obese had normal height for age.

### Serum micronutrient status

The serum micronutrient status of the children is shown in Table 3. Indicators for iron status namely, ferritin and haemoglobin concentrations were found to be adequate in the majority of the children. Only 2.2% had unsatisfactory concentrations for either indicator, with only one case of iron deficiency anaemia. Anaemia can also result from deficiencies of other micronutrients including zinc, vitamin B_12_, and folate. However, almost all the children in this study had normal zinc concentrations, whilst all had normal vitamin B_12 _and folate concentrations. None of these serum micronutrient results showed significant differences between the boys and girls.

**Table 3:** Serum micronutrient status of the children

	Boys	Girls	Total	*P value
	
		N (%)		
**Ferritin (μg/L)**				0.313
Low (< 12)	2 (1.1)	7 (3.2)	9 (2.2)	
Normal (12 - 140)	174 (97.2)	213 (95.9)	387 (96.5)	
High (> 140)	3 (1.7)	2(0.9)	5 (1.2)	
Total	179 (100)	222 (100)	401 (100)	
**Haemoglobin (g/L)**				
Low (< 110)	1 (0.6)	8 (3.6)	9 (2.2)	0.080
Normal (110 - 150)	179 (99.4)	213 (95.9)	392 (97.5)	
High (> 150)	0	1 (0.5)	1 (0.2)	
Total	180 (100)	222 (100)	402 (100)	
**Mean Corpuscular Volume (MCV) (fL)**				
Low (< 75)	21 (11.7)	37 (16.7)	58 (14.4)	0.156
Normal (75 - 93)	159 (88.3)	185 (83.3)	344 (85.6)	
Total	180 (100)	222 (100)	402 (100)	
**Iron Deficiency Anaemia**				
(Ferritin <12 μg/L + Hb <110 g/L + MCV <75 fL)	1 (0.6)	0	1 (0.2)	-
	180 (100)	222 (100)	402 (100)	
**Serum Zinc (μmol/L)**				
Low (< 9.0)	1 (0.6)	4 (1.8)	5 (1.2)	0.458
Normal (9.0 - 18.0)	177 (98.3)	217(97.7)	394 (98.0)	
High (> 18.0)	2 (1.1)	1 (0.5)	3 (0.7)	
Total	180 (100)	222 (100)	402 (100)	
**Vitamin B_12 _(ρmol/L)**				
Normal (> 185)	179 (100)	222 (100)	401 (100)	-
**Folate (nmol/L)**				
Normal (> 5.7)	179 (100)	222 (100)	401 (100)	-
**25-hydroxyvitamin D (nmol/L)**				
Deficiency (≤37.5)	51 (28.3)	91 (41.0)	142 (35.3)	0.010
Insufficiency (> 37.5 - 50.0)	68 (37.8)	81 (36.5)	149 (37.1)	
Sufficiency (> 50.0)	61 (33.9)	50 (22.5)	111 (27.6)	
Total	180 (100)	222 (100)	402 (100)	

*Pearson χ^2 ^*p *value, with significance *at P *< 0.05

References for classification of serum micronutrients:

Haemoglobin and Mean Corpuscular Volume: [9]

Ferritin: [10]

Zinc: [11]

Vitamin B_12 _and Folate: [12]

Vitamin D: [13])

In contrast, there was a high prevalence of vitamin D insufficiency, with 70.4% having 25(OH)D concentrations <50 nmol/L. Out of this, there were almost equal proportions of the children with vitamin D deficiency (35.3% having ≤37.5 nmol/L) and insufficiency (37.1 > 37.5 < 50.0 nmol/L). Moreover, there was a significant association (*P *= .010) between vitamin D status and sex, there being a higher prevalence of vitamin D insufficiency among the girls (77.5%) than boys (66.1%)

Further analysis of the association between vitamin D status and BMI-for-age revealed a significant trend only for the boys (χ^2 ^= 5.958; *P *= .016) (Table 4). While the proportion of overweight boys (17.2%) was close to that among the girls (18.5%), the proportion of obesity in boys was more than two-fold that of the girls (25% and 9.5% respectively).

**Table 4:** Correlation between BMI-for-age and vitamin D status

	**Boys (n = 180)**				**Girls (n = 222)**				**Total (n = 402)**			
	
	**Vitamin D status**				**Vitamin D status**				**Vitamin D status**			
	
**BMI-for-age Status**	*Low		**Normal		*Low		**Normal		*Low		**Normal	
			
	**N**	**%**	**N**	**%**	**N**	**%**	**N**	**%**	**N**	**%**	**N**	**%**
			
Severe thinness	3	2.5	2	3.3	1	0.6	0	0.0	4	1.2	2	3.4
Thinness	6	5	5	8.2	10	5.8	4	8.0	20	5.8	5	8.6
Normal	54	45.4	34	55.7	109	63.4	36	72.0	194	56.4	39	67.2
Overweight	20	16.8	11	18.0	34	19.8	7	14.0	66	19.2	6	10.3
Obese	36	30.3	9	14.8	18	10.5	3	6.0	60	17.4	6	10.3
*x*2 (df = 1)	5.958				1.160				5.832			
p-trend (Monte Carlo)	0.016				0.330				0.013			

* Low vitamin D concentration: ≤50 nmol/L

** Normal vitamin D concentration: >50 nmol/L

## Discussion

In general, the nutritional status of the children attending primary schools in Kuala Lumpur was good with respect to height-for-age and adequacy in several micronutrients examined. These results may be due to the socio-demographic background of the families - most parents having formal education, earning middle level income on average, and with access to affordable health care and exposure to nutrition information in the capital city of Kuala Lumpur.

The prevalence of underweight was lower than previously reported [7]. Nearly all the children (96.6% of boys and 97.3% of girls) had normal height-for-age. A child with normal bodyweight for age is more likely to become overweight or obese if he or she is stunted; however, this was not the case in this study as the prevalence of stunting was low, at less than 5%. This finding is encouraging compared to previous studies. Zalilah *et al*., (2000) reported that as high as 50% of primary school children in Kuala Lumpur were stunted, in a survey of 4212 boys and 3793 girls aged 6-10 years, albeit from low income households [16].

This study however found a high prevalence of obesity in the young children. More than one-third of the children aged 7-12 years were overweight or obese based on BMI-for-age. This finding is higher than that reported by previous studies on children in Kuala Lumpur city. In 2002, Tee *et al*., [17] reported that 8.4% of primary school children (n = 5,995) in Kuala Lumpur were overweight, based on the WHO 1995 definition [18], while Moy *et al*., (2004) [19] reported 10.1% were overweight, based on BMI-for-age >95^th ^percentile, among 1,320 schoolchildren in the capital city. Differences in definition and criteria used by the various studies may explain some of the different results on excess adiposity in the urban children studied. Notwithstanding that, the high overweight and obesity prevalence in the present study is a matter of public health concern.

The results on iron status were encouraging since moderate to high levels of anaemia have long been one of the predominant findings of nutrition surveys among children in Malaysia [20,21]. The biochemical results also indicated that none of the children showed deficiency of zinc, folate and vitamin B_12_. These findings may reflect adequacy in their dietary intake. Based on self-reported dietary history (data not shown), the intake of the children for several nutrients including protein, iron, vitamin A, thiamine, riboflavin and vitamin B_12 _were better than the recommended nutrient intakes (RNI) for Malaysian children [22].

The importance of this study is the unexpected finding of a large proportion of the children showing vitamin D deficiency (35.3%), and an almost similar proportion with insufficient vitamin D status (37.1%). We did not expect apparently healthy school children to show vitamin D deficiency, given that Kuala Lumpur is located at latitude 03° 09'N, with almost all year round of UV-B radiation of sufficient wavelength necessary for cutaneous synthesis of vitamin D. Indeed, at a recent seminar, it was revealed that cases of rickets have been presented in paediatric wards in Malaysia in recent years [23]. There are increasing studies world-wide showing children with poor vitamin D status, including those in tropical countries [24-28].

Poor vitamin D status in children is likely to result from low dietary intake and inadequate exposure to sunshine. Unless fortified, most foods are poor sources of vitamin D, exceptions being fish oils, egg yolk, and certain types of fish and sea food. Few foods in Malaysia are fortified with vitamin D, and these are confined to some margarines and beverages only. Thus, it is not likely that children in Malaysia will obtain sufficient vitamin D from dietary sources alone.

Based on self-reported daily sunlight exposure in this study, the children also seem not to be receiving enough sun exposure, as they tend to spend more time indoor than outside the house. Moreover, indoor activity is sedentary in nature e.g. doing homework, playing computer games and watching television. Malaysians generally avoid being outdoor during the day as the weather can be very hot and humid. Moreover, parents may be concerned about safety and may not allow their children to play outside the home unaccompanied. Also, the type of clothing worn due to culture or religion may limit the capacity of the skin to synthesize vitamin D. Some of these behaviours are likely to be more evident in girls than boys, and this may explain in part the finding that more girls were deficient in vitamin D than boys. Similar findings about limited sun exposure and low dietary intake have been reported for children in tropical countries [29,30].

Another key finding of this study is the inverse relation between vitamin D status and BMI-for-age. The boys in this study, being more obese than the girls on average, were found to have a significant negative association with vitamin D status (χ^2 ^= 5.958; *P *= .016). Other studies have also shown obesity to be associated with decreased vitamin D status in children [31,32]. Vitamin D, being fat-soluble, is readily stored in adipose tissue, and thus, it could be sequestered in the larger body pool of fat of obese individuals. As a result, there is reduced bioavailability and lower serum vitamin D concentrations [33,34]. This has raised the concern that in obese individuals, serum 25(OH)D may not be a good indicator of vitamin D stores.

Results of poor vitamin D status amongst Malaysian school children deserve public health attention. There is the need to dispel misconceptions that low vitamin D status does not occur in a tropical country like Malaysia. Parents and teachers should be provided with information on the importance of vitamin D in the growth and development of children. In addition to helping parents make the correct dietary choices to improve vitamin D status of their children, there is a need to encourage growing children to gain adequate sun exposure through physical activity outdoors in school and at home. Public health messages and interventions must educate the population about safe sun exposure, since UV-B radiation of the same wavelength (290-315 nm) necessary to stimulate endogenous vitamin D synthesis, also contributes to skin cancers, as excess exposure causes DNA damage and skin burning.

### Limitations of study

In this study, a cut off of ≤ 37.5 nmol/L was used to derive the extent of vitamin D deficiency and 37.5 - 50.0 nmol/L to indicate insufficiency. Although there is no global consensus on the biochemical definition of vitamin D deficiency, a cut-off point of ≤ 37.5 nmol/L (≤ 15 ng/mL) 25(OH)D is typically used to determine deficiency [13].

As the original objective of the study was to assess the micronutrient status of children, parathyroid hormone (PTH) was not determined. PTH determination should be included as a functional index of vitamin D status in future studies. It is also important to use objective measures to determine sun exposure, dietary intake and physical activity. A direct measure of fat mass, beside body mass index, may provide a more sensitive relationship between vitamin D concentration and obesity in children.

## Conclusions

The nutritional status of Malaysian children in general has improved substantially over recent decades in tandem with rapid socioeconomic development and substantial investment in public health improvement efforts. This study revealed that while, the primary school children in Kuala Lumpur city had adequate serum iron, folate, zinc and vitamin B_12 _concentrations, the majority of them had sub-optimal concentrations of serum vitamin D. In addition, the finding of an inverse association between vitamin D status and obesity raises concern as the prevalence of obesity in children is on the rise in Malaysia. More innovative approaches are needed to encourage more children to be actively involved in outdoor sports and games towards addressing both the problem of childhood vitamin D insufficiency and obesity.

## Competing interests

The authors declare that they have no competing interests.

## Authors' contributions

GLK, WSSC, ZMS, BKP and HET conceived and designed the study. GLK, WSSC, ZMS, BKP & MA conducted the field work. JAR assisted in statistical analysis. All authors read and approved the final manuscript.

## Pre-publication history

The pre-publication history for this paper can be accessed here:

http://www.biomedcentral.com/1471-2458/11/95/prepub
